# Severe Dental Disease as a Presenting Sign of Relapsed 6q24-Related Transient Neonatal Diabetes Mellitus

**DOI:** 10.1155/2020/8828516

**Published:** 2020-11-16

**Authors:** Anna Delamerced, Lauren J. Massingham, Jose Bernardo Quintos

**Affiliations:** ^1^The Warren Alpert Medical School of Brown University, Providence, RI, USA; ^2^The Warren Alpert Medical School of Brown University, Rhode Island Hospital, Hasbro Children's Hospital, 593 Eddy Street, Providence, Rhode Island 02903, USA

## Abstract

Transient neonatal diabetes mellitus (TNDM) is a rare form of diabetes that presents in infancy and is characterized by intrauterine growth restriction and hyperglycemia without ketones on urinalysis. Patients are treated with insulin until remission, usually within the first year. Relapse to a permanent state may occur later in life, with a mean age of 14 years. The most common cause of TNDM is a chromosome 6q24 mutation that affects pancreatic *β*-cell function. Reports of relapse have been limited. We describe a case of an adolescent female with TNDM due to 6q24 hypomethylation who relapsed at 15 years of age with severe dental disease as the presenting sign.

## 1. Introduction

Transient neonatal diabetes mellitus (TNDM) associated with 6q24 chromosomal aberrations is a rare type of diabetes that presents in the neonatal period within the first six weeks of life [1]. Suggestive findings include severe intrauterine growth restriction (IUGR), hyperglycemia, dehydration, and lack of ketoacidosis [1]. When insulin is no longer needed, remissions occur during infancy by 18 months of age, with the mean age of remission at 4.5 months of age [2]. Patients can relapse in late childhood, adolescence, or adulthood [3, 4]. 6q24-related TNDM occurs in approximately 1 in 400,000 live births [5].

There are more than 20 known genetic causes. The most common cause of TNDM, in approximately 70% of affected patients, is a mutation in chromosome 6q24 that affects pancreatic *β*-cell function. Diagnosis is established with genetic testing [1].

The mean age of relapse is 14 years, although relapse has been reported to occur as early as at 4 years of age [2]. Relapsed TNDM is characterized by decreased insulin secretion, lack of autoantibodies, and no obesity. Approximately 50% of patients with 6q24-related TNDM may relapse, with no standard treatment for relapse [6, 7]. There are few case reports documenting relapse presentation in 6q24-related TNDM.

We describe a case showing severe dental disease as the primary presenting sign of relapsed 6q24-related TNDM. Initial clinical presentation, presentation at relapse, workup, and management are discussed.

## 2. Case Presentation

The patient is a 19-year-old female with a history of 6q24-related TNDM and Hashimoto's thyroiditis who was diagnosed with neonatal diabetes mellitus at birth.

She was born to a 25-year-old G3P2 mother, with a weight of 1927 grams, at 36 weeks of gestation via vaginal delivery after induction due to IUGR at an outside hospital. The pregnancy was significant for intrauterine growth restriction noted at 5 months of gestation. The pediatrician was at the delivery, and no resuscitation was needed. Apgar scores were 8 at 1 minute and 9 at 5 minutes. Macroglossia was noted. Umbilical hernia and hypotonia were not found. The infant was noted to have hypoglycemia on day of life one and was given intravenous glucose and went on to develop hyperglycemia. Glucose levels were the 400 mg/dl, and an insulin drip was started. The infant was transferred to the NICU from the outside hospital due to hyperglycemia and concerns for necrotizing enterocolitis (NEC). Due to concerns for NEC, oral feeds were discontinued and she was placed on TPN, providing a steady glucose load. No surgical intervention was necessary for the NEC. Glucose was difficult to control even while on TPN with blood sugar levels ranging from the 100s to >200 mg/dl. At about 1 month of age, glucose levels started dropping and insulin was weaned. During this time, due to an IV infiltrate, IV insulin was briefly discontinued and blood glucose was 268 mg/dl. Insulin infusion was restarted and was discontinued at about 1 month of age. Blood sugar levels ranged between 80 and 150 mg/dl. The infant was discharged shortly afterward, feeding ad lib and checking blood sugar before meals. She required intermittent subcutaneous insulin at home, which was finally discontinued at approximately 4 months of age. Since then, the patient did not require insulin or have diabetes symptoms. All developmental milestones were on time. At 15 years of age, she chipped her tooth and went to the dentist for evaluation. The dentist noted extensive tooth decay. Jaw X-rays showed bone loss of nearly 80% in the left mandible. Due to her history of TNDM, she was sent to her primary care provider for further workup and diabetes screening where blood glucose was 369 mg/dl and urinalysis showed positive ketones. She was sent to the emergency department, and laboratory examination showed blood glucose of 361 mg/dl, Na+ of 131 mEq/L, *K*+ of 3.4 mmol/L, bicarbonate of 19 mmol/L, 2+ urine ketones, anion gap of 16, and pH of 7.4. HbA1c was 15.4% during her admission. The diabetes autoimmune panel was negative (ICA-512, GAD-65, and Insulin Antibodies).

She endorsed long-standing polydipsia, polyphagia, and polyuria. She denied weight loss, nausea, vomiting, abdominal pain, confusion, blurry vision, fatigue, fever, and difficulty breathing. Family history was negative for autoimmune diseases except for hypothyroidism in the paternal grandmother and maternal great-grandmother. Physical exam showed a weight of 58.4 kg (70th percentile), height of 163 cm (55^th^ percentile), and BMI of 21.98 kg/m^2^ (70th percentile). She was a well-appearing adolescent without thyromegaly and acanthosis nigricans and had Tanner Stage 5 breast. She was started on basal bolus insulin regimen consisting of 23 units of Glargine at bedtime and fast-acting insulin Lispro 1 unit per 10 grams of carbohydrates with meals.

She received the appropriate diabetes education. Laboratory examination also noted a TSH of 66 IU/ml (reference range: 0.35–5.5 uIU/ml); free T4 was 0.94 ng/dl (reference range: 0.8–1.8 ng/dl), and positive antibodies to thyroid peroxidase was 8580 IU/ml. She was treated with 75 mcg Levothyroxine that was titrated to a dose of 100 mcg. The 6q24 methylation-specific multiplex ligation-dependent probe amplification (MPLA) genetic test for transient neonatal diabetes identified hypomethylation within the 6q24 region. Deletion/duplication analysis ruled out paternal duplication of 6q24. Therefore, the hypomethylation could be the result of either paternal uniparental disomy of chromosome 6 (UPD6) or hypomethylation of the maternal allele. Via testing, UPD6 has been confirmed, meaning both copies of the 6q24 chromosome region in our patient were paternally inherited. This results in an epigenetic phenomena where both copies are still active. Typically, the paternal copy is active and the maternal copy is methylated and turned off. Epigenetic changes are typically de novo, as they depend on the parent of origin and are reset with each pregnancy, rather than DNA sequencing changes. Thus, risk of recurrence for parents, siblings, and offspring is unlikely. After diagnosis and her initial HbA1c of 15.4%, her HbA1c has ranged since then from 6.3% to 6.8%.

## 3. Discussion

We present a 19-year-old female with a history of TNDM who presented with neonatal diabetes mellitus in the newborn period, went into remission at 4 months of age, and presented with severe dental disease when she relapsed at 15 years of age. To the best of our knowledge, there have been no case reports of severe dental disease as the initial presenting sign of 6q24-related TNDM relapse.

Clinical features, management, and prognosis are influenced by the gene involved. The majority of TNDM cases is caused by a mutation in chromosome 6q24 that affects pancreatic *β*-cell function. 6q24-related TNDM can be caused by three mechanisms: paternal uniparental disomy of chromosome 6, duplication of the paternal 6q24 allele, or loss of maternal methylation [8].

Initial presentations of TNDM have been documented and usually involve hyperglycemia and excessive dehydration, within the first week of life, which gradually resolves between 3 and 18 months. In particular, 6q24-related TNDM is characterized by low birth weight and intrauterine growth restriction. One study found that 30% of 6q24-related TNDM patients were born at the time of less than 37 weeks of gestation [2]. The low birth weight is likely due to the failure of insulin secretion, which is important for intrauterine growth [1]. Additional characteristics that may present in patients include an umbilical hernia, macroglossia, or hypotonia. Less frequently reported features include renal and cardiac abnormalities [2].

Data on 6q24-related TNMD relapse are scarce. Presenting symptoms of relapse have not been documented extensively. Relapse may be due to increased physiologic stress or increased insulin demand, including childhood illness, the onset of puberty, or pregnancy. The median age of TNDM relapse is 14 years, but the age and presenting signs of relapse vary widely and can occur as early as at 4 years of age or as late as 27 years of age [3, 9]. Only a few cases of 6q24-related TNDM have been reported and were diagnosed incidentally or by routine laboratory investigation. These cases of relapsed diabetes were incidentally discovered after a routine glucose test (Table 1).

Our patient presented with a unique finding of severe dental disease as the main clinical presentation for relapsed 6q24-related TNDM. No previous cases have reported severe dental disease, gum disease, dental caries, or bone loss of the jaw as primary features for TNDM relapse. An elevated TSH and positive anti-thyroid peroxidase antibodies, consistent with acquired hypothyroidism, could have contributed to bone loss of the jaw, as thyroid hormones play a role in bone mineral density.

After relapse, some patients have transitioned to oral hypoglycemic agents. While sulfonylureas have been used to treat relapsed TNDM, no specific guidelines exist for management of relapse. Possible treatments include targeting the GLP-1 pathway because these patients still have the ability to produce and respond to insulin through *G* protein-coupled pathways [6]. We plan to transition our patient to an oral hypoglycemic agent and to wean off and discontinue insulin.

In summary, little is known of the clinical presentation for 6q24-related TNDM relapse. We present a patient who was diagnosed with neonatal diabetes mellitus as an infant, went into remission at 4 months of age, and relapsed at 15 years of age with dental caries as the primary characteristic. Accumulation of further cases is needed to increase knowledge of management of 6q24-related diabetes relapse with insulin or hypoglycemic agents.

## Figures and Tables

**Table 1:** Review of the literature of 6q24-related TNDM at relapse.

Reference	Age of relapse	HbA1c or fasting glucose level at relapse	Genetic variant	Treatment
Schimmel [10]	15 years	198 mg/dl	Uniparental paternal isodisomy of chromosome 6	Sulfonylurea

Yorifuji et al. [7]	12 years	7–7.5%	Paternal duplication at 6q24	Initially alpha-glucosidase inhibitor, then DPP4-inhibitor

Fu et al. [11]	14 years	8.2%, 204 mg/dl	Hypomethylation of the maternal allele	Insulin glargine and lifestyle modifications, transitioning to sulfonylurea

## Data Availability

No data were used to support this study.
